# Docking in the *Abies grandis* abietaenol synthase to investigate the mechanism underlying conifer resin acid diterpene synthase evolution

**DOI:** 10.1042/BCJ20250294

**Published:** 2026-02-24

**Authors:** Mark Schmidt-Dannert, Griffin Humphreys, Taylor Eich, Meirong Jia, Reuben J. Peters

**Affiliations:** 1 Roy J. Carver Department of Biochemistry, Biophysics & Molecular Biology, Iowa State University, Ames, IA 50011, U.S.A.; 2 State Key Laboratory of Bioactive Substance and Function of Natural Medicines, NHC Key Laboratory of Natural Products, CAMS Key Laboratory of Enzyme and Biocatalysis of Natural Drugs, Institute of Materia Medica, Chinese Academy of Medical Sciences and Peking Union Medical College, Beijing 100050, China

**Keywords:** diterpenoid, enzymology, evolutionary biology

## Abstract

The pine family (Pinaceae) produces oleoresins in which the diterpenoid resin acids contain tricyclic backbones of either the (iso)pimarane and/or rearranged abietane type. These are produced by closely related (class I) diterpene synthases, whose functional divergence is of significant interest, not least for mechanistic insights into the catalyzed complex carbocation-cascade reactions. The abietaenol synthase from *Abies grandis* (AgAS) has long served as a model for this enzymatic subfamily and was used here to examine key residues potentially involved in transitions from such ancestral activity to production of (iso)pimaradienes, which seems to have occurred independently at least three times in the Pinaceae. While the equivalent substitutions in AgAS led to production of some amount of isopimaradiene(s), based on the transition observed in the most closely related enzymes (i.e., those also from the *Abies* genus), the threonine substitution for an alanine was most impactful, with the resulting A723T variant producing almost entirely isopimara-7,15-diene. This prompted application of the *TerDockin* computational approach to investigate the underlying mechanism. Through iterative application, requiring placement and constraint of the reactant water from the native reaction (i.e., that added to yield the 13α-hydroxyl of the primary abietaenol epimer), it was found that the introduced threonine most likely acts directly as a catalytic base to short-circuit the native reaction by deprotonating the initially formed isopimara-13*E*-en-8-yl carbocation intermediate. These results not only provide mechanistic insight but also have some implications for such modeling of the complex reactions catalyzed by terpene synthases more generally, as discussed herein.

## Introduction

Terpene synthases catalyze carbocation cascade reactions, often forming complex stereo-dense hydrocarbon backbones, terminated by deprotonation, which can be preceded by the addition of water to yield a hydroxyl group rather than an olefin [1]. Given the inherent reactivity of carbocations [2], even unactivated hydroxyl groups can serve as the relevant catalytic base [3]. Notably, the terpene synthase-catalyzed reaction represents a key, if not the committed, step in most terpenoid biosynthetic pathways [4]. In the case of the diterpenoid resin acids found through the Pinaceae, this is the formation of either (iso)pimarane or abietane-type olefin precursors from copalyl pyrophosphate (CPP, **1**) [5]. The relevant diterpene synthases are from the gymnosperm-specific terpene synthase subfamily TPS-d, more specifically the TPS-d3 group [6]. Strikingly, while each of the three major genera, fir (*Abies*), pine (*Pinus*), and spruce (*Picea*), produces both abietanes and (iso)pimaranes, the relevant (iso)pimaradiene synthase (PS) clearly evolved in parallel from an ancestral abietaenol synthase (AS) via gene duplication and neo-functionalization in each lineage [5].

The first such diterpene synthase to be identified was that from *Abies grandis* (AgAS) [7]. This is a bifunctional enzyme also containing a diterpene cyclase that first converts the general diterpenoid precursor geranylgeranyl pyrophosphate (GGPP) to **1**. The diterpene synthase active site in AgAS was originally proposed to selectively convert **1** to abietadiene (abieta-7,13-diene, **2**) [7]. It was later suggested to produce a mixture of abietane olefin isomers—i.e., abietadiene, neoabietadiene (abieta-8(14),13(15)-diene, **3**), palustradiene (abieta-8,13-diene, **4**), and, despite the name, levopimaradiene (abieta-8(14),12-diene, **5**) [8]. However, it has since been proposed that these arise from the facile dehydration of abietaenol (abieta-8(14)-en-13-ol, **6**), the carbon-13 (C13) epimers of which are then the primary product of the Pinaceae abietane-type diterpene synthases more generally (Supplementary Figure S1) [9]. Regardless, its early discovery led to extensive studies with AgAS. Among these were labeling studies identifying the initially formed intermediate as containing the 13β-methyl configuration—i.e., this is isopimara-15-en-8-yl carbocation (**A**)—resulting from anti S_N_’ cyclization upon initiating ionization of the allylic pyrophosphate ester in **2** [10]. Further studies with substrate and intermediate analogs indicated **A** undergoes ring inversion and substantial rotation of the vinyl group to enable an intramolecular 1,4-proton transfer. This is driven at least in part by carbocation migration towards the pyrophosphate anion co-product. Subsequent further rotation is required to enable a suprafacial 1,2-methyl shift to form the isopropyl substituent that characterizes abietanes, resulting in the concluding abieta-8(14)-en-13-yl carbocation intermediate (**B**) [11].

**Figure 1:**
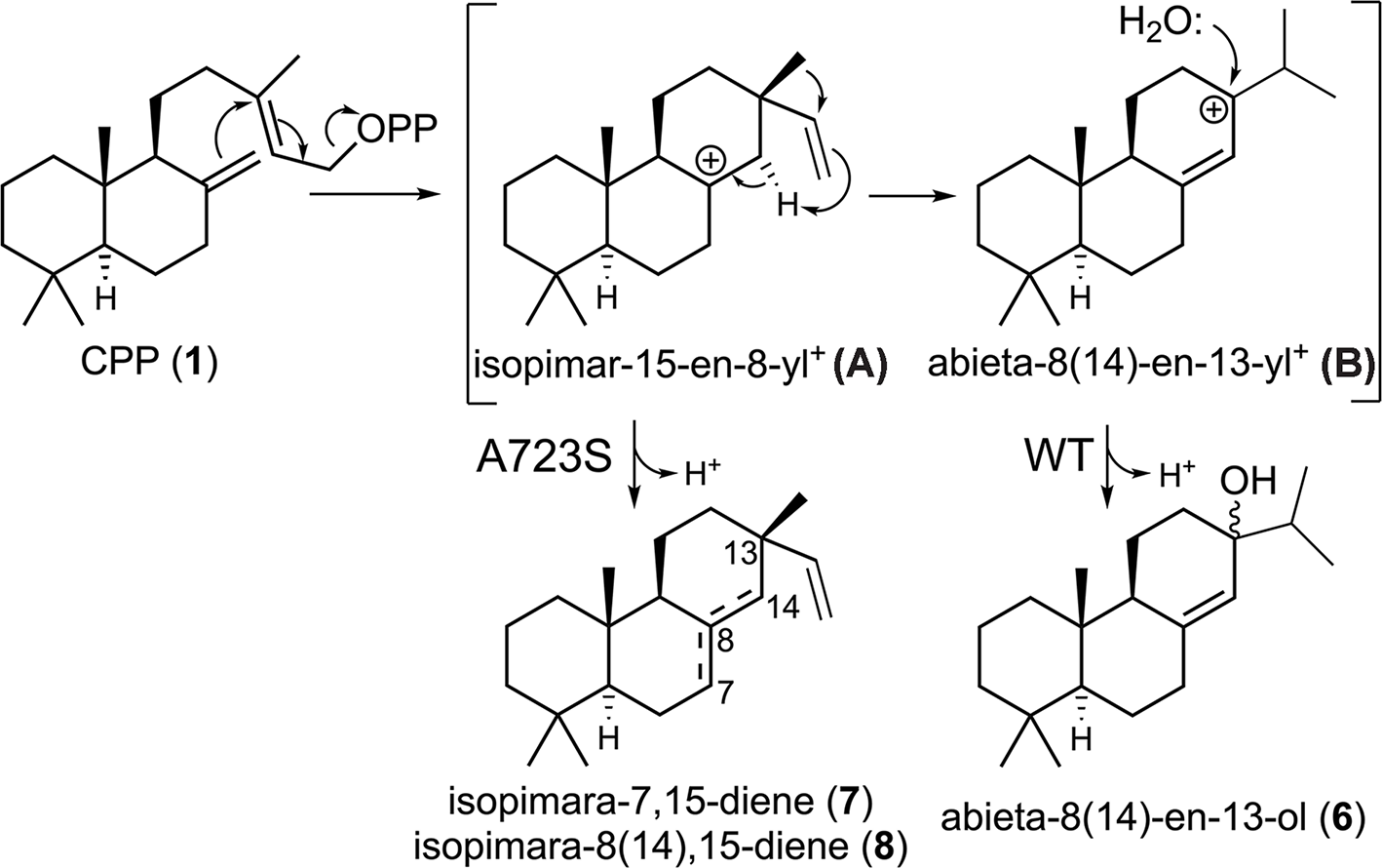
AgAS catalyzed reaction and known effect of A723S substitution. Reaction catalyzed by AgAS (diterpene synthase active site) and previously reported effect of A723S mutation [14].

Given the matching configuration between **A** and the isopimara-7,15-diene (**7**) precursor for the commonly observed isopimaric acid constituents of Pinaceae resins, which then simply represents deprotonation of **A**, it is perhaps not surprising that the relevant PS identified from *Picea abies* (PaPS) was closely related to its AS (PaAS), sharing >90% amino acid sequence identity [12]. Building on the discovery of a single residue isoleucine to threonine switch that leads to analogous short-circuiting (i.e., deprotonation of a pimarenyl carbocation intermediate) in a similarly otherwise more extended reaction in plant *ent*-(iso)kaurene synthases [13], sequence comparison identified a nearby residue that is conserved as alanine in AgAS and PaAS but is a serine in PaPS. Indeed, substitution of serine for this alanine in AgAS led to predominant production of **7**, along with a small amount of the olefinic isomer sandaracopimaradiene (isopimara-8(14),15-diene, **8**) by the resulting A723S variant (Figure 1) [14]. A similar effect of such substitution was observed with PaAS, although it must be noted that additional changes were required to fully exchange specific product outcomes between PaPS and PaAS, indicating epistatic effects most likely due to subtle changes in reactant positioning [15]. While it has been suggested that the hydroxyl group introduced by serine substitution might act via an indirect electrostatic effect [16], later resolution of a crystal structure for AgAS revealed that A723 sits at the G1/G2 helix break, with its side chain protruding into the active site cavity [17]. This closely resembles the previously reported use of the hydroxyl introduced in an analogous serine for alanine substitution as a catalytic base to deprotonate a similar *ent*-pimara-15-en-8-yl^+^ intermediate in a bacterial *ent*-kaurene synthase [3] and suggests the hypothesis that the A723S mutational effect on AgAS product outcome also might be attributed to such a direct mechanism.

Here, sequence comparison is utilized to identify alternative potential changes for product outcome in AgAS, with identification of an A723T substitution, based on the more closely related AS and PS pair from *Abies balsamea* [18], leading to an essentially complete shift from production of **5**, as verified here, to production of **7**. In turn, this led to the application of the *TerDockin* computational approach, applied in an iterative fashion to not only provide insight into the native reaction mechanism but also evidence supporting direct catalytic activity for the hydroxyl introduced by either serine or, especially, threonine acting as the base for deprotonation of **A** to yield **7** (e.g., by AgAS:A723T variant), with the implications of these studies then discussed.

## Results and discussion

### Identification of conservation patterns

**Figure 2:**
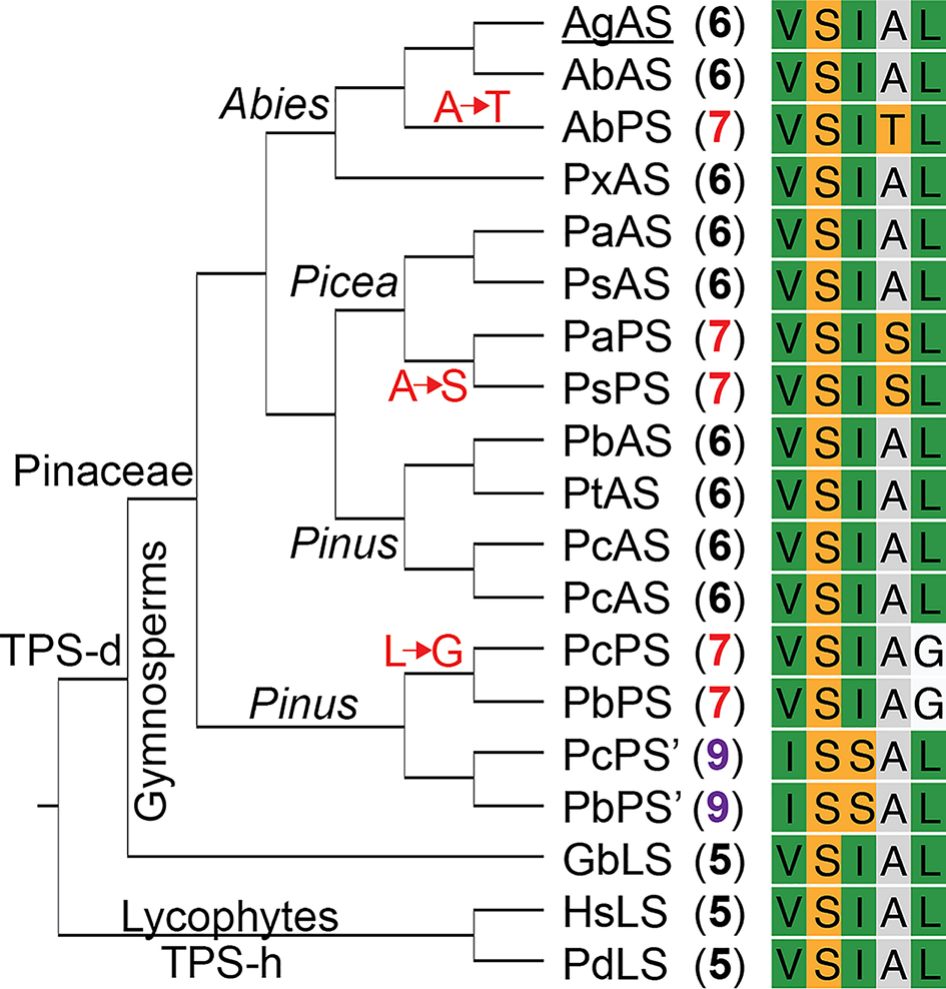
Conservation of key VSIAL motif in gymnosperm resin acid and related diterpene synthases. Conservation of the VSIAL motif in AS (and LS) with observed differences in PS as indicated by phylogenetic tree (cladogram labeled with genus/family and relevant TPS subfamilies), associated sequences (and key residue changes), and product (numbered as described in text, pimaradienes are colored with red indicating (iso) C13 configuration relevant to abietaenol production, while purple is epimer).

While conservation of the key alanine between AgAS and PaAS was previously noted [14], it has since been realized this is more broadly conserved within the abietane-producing TPS-d3 subfamily members [18–22], including one producing predominantly levopimaradiene [23], Notably, this alanine is embedded in a VSIAL motif that may have arisen even before the gymnosperms, as two levopimaradiene synthases (LSs) from lycophytes, which are then members of the TPS-h subfamily (albeit the two most closely related to the TPS-d subfamily), also contain this sequence [24]. While residues in this motif vary in the (iso)pimaradiene-producing members of the TPS-d3 subfamily members, it must be noted that serine substitution of the alanine to drive production of isopimara-7,15-diene (**7**) only seems to have occurred within the *Picea* (Figure 2). Indeed, gene duplication of an ancestral AS with subsequent neofunctionalization of one copy to production of **7** seems to have occurred independently in each of the main genera—i.e., *Abies*, *Picea*, and *Pinus* [25]. Nonetheless, the PS from *A. balsamea* may have utilized a similar threonine substitution instead (i.e., threonine is found in place of the alanine in AbPS) [18]. By contrast, the (iso)pimaradiene synthases from *Pinus* (both *P. banksiana* and *P. contorta*) still contain the alanine but have other deviations from the VSIAL motif, with those producing **7** containing glycine in place of the leucine (i.e., VSIAG), while those producing the 13-epimer (α-methyl) pimara-8(14),15-diene (**9**) contain two differences—i.e., ISSAL [21].

### Product analysis

**Figure 3:**
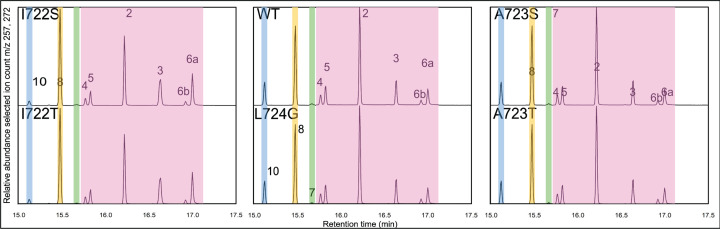
Effect of phylogenetically guided subsitution for VSIAL motif in AgAS. GC-MS chromatograms of the product outcomes mediated by the indicated AgAS variants *ex vivo* (peaks numbered as described in the text, with colored shading by origin/identity—i.e., pink indicates a mixture of abietanes derived from dehydration of **6** during analysis).

Upon co-expression with a GGPP synthase in *Escherichia coli*, extraction of the resulting culture with organic solvent provides access to the resulting products, providing an *ex vivo* assay for changes in product outcome [26], which was applied here to AgAS. Notably, the GC-MS chromatogram for wild-type (WT) AgAS contains the epimeric pair of abietaenol peaks, alongside the previously reported olefins, in similar amounts as previously reported for other ASs (see Figure 3 and Supplementary Figure S2) [18,20–22]. Thus, AgAS also appears to be an AS. Given the uncertainty in the previously reported structural assignment [9], the AgAS products were purified, and NMR analysis was carried out for both epimers (Supplementary Tables S1 and S2 and Supplementary Figures S3–S16). This revealed the major product (∼70%) to be the α-hydroxy epimer (abieta-8(14)-en-13α-ol, **6a**), with the β-epimer (abieta-8(14)-en-13β-ol, **6b**) also found in smaller amounts (∼30%), with both sets of assigned chemical shifts supported by computational prediction [27].

### Mutational analysis

To investigate the observed correlation between product outcome and alterations in the VSIAL motif, analogous substitutions were made in AgAS, along with an additional threonine substitution for the isoleucine to match the pair of neighboring alanine substitutions. In addition to the previously reported A723S variant [14], this entailed construction of I722S, I722T, A723T, and L724G variants. Each of these was then examined via the *ex vivo* assay noted above (Figure 3). As previously reported [14], AgAS:A723S produces primarily **7** (58%) alongside smaller amounts (15%) of the olefinic isomer sandaracopimaradiene (**8**), as well as **6** (27%, computed from the sum of the peak areas of **1**–**6**). Notably, AgAS:A723T exhibits an essentially complete product switch to **7** (97%), with very little **8** and no **6** observed. While the effectiveness of this single residue switch is perhaps not surprising, given its basis on the analogous change observed between the more closely related AS and PS from another *Abies* species (i.e., AbAS and AbPS; see Figure 2), it should be noted that substitution of serine for the relevant alanine in the *Picea* PaAS, matching that found in the paralogous and closely related PaPS (Figure 2), was not nearly as effective [15].

Substitution of serine and, especially, threonine for the isoleucine (I722S/T) also leads to changes in product outcome. While AgAS:I722S still produces primarily **6** (88%), appreciable amounts of **8** (7%), as well as the olefinic isomer isopimara-8,15-diene (**10**) (5%), are also observed. Notably, AgAS:I722T exhibits a much stronger effect on product outcome, with significant amounts of **8** (35%) observed along with **6** (63%). Substitution of glycine for the leucine also shows a marked effect on product outcome, with AgAS:L724G producing substantial amounts of **8** (29%), along with appreciable amounts of **10** (7%), although the primary product is still **6** (64%). Given the much more distant relationship of the *Pinus* PS where serine is found in place of the isoleucine, not to mention the difference in configuration (C13 epimer—i.e., α-methyl), the marked decrease in effect on AgAS product outcome is perhaps unsurprising. Intriguingly, despite not having been phylogenetically observed, threonine substitution shows a greater effect on product outcome. However, there is no change in C13 configuration, just deprotonation of isopimara-15-en-8-yl^+^ (**A**) at alternative positions (primarily C14). Nonetheless, this suggests that the methyl group substituent distinguishing threonine from serine, at both positions 722 and 723, may be important in positioning **A** for deprotonation, albeit at differing carbons (C14 versus C7, for production of **8** versus **7**, respectively). By contrast, in the *Pinus* PS where glycine is found in place of leucine, these primarily produce **7**, but the effect of such substitution in AgAS is to increase production of **8** instead. Regardless, this also indicates differences in positioning of **A** in AgAS relative to these *Pinus* PSs.

### TerDockin analysis

**Figure 4:**
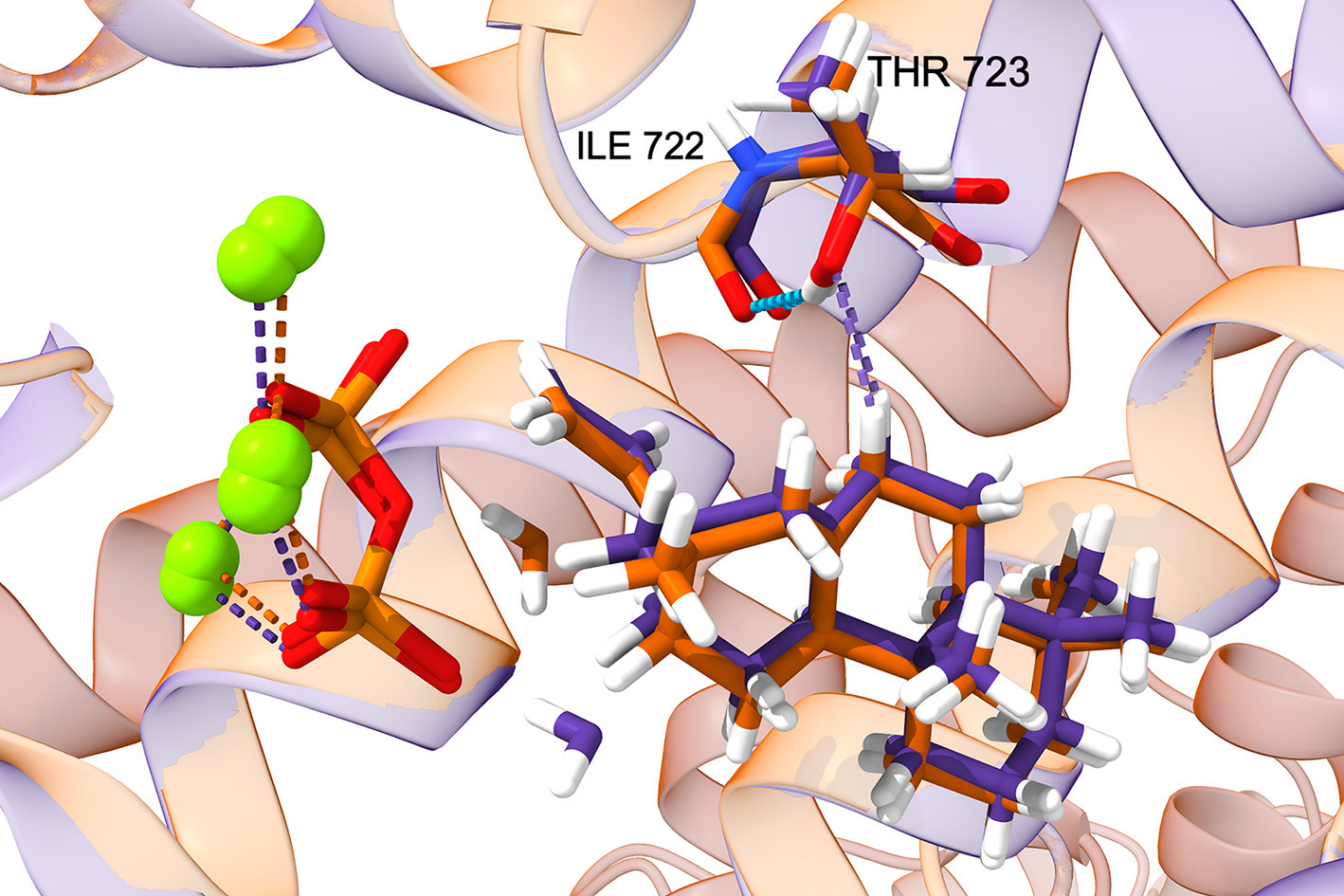
*TerDockin* results for AgAS:A723T including "reactant" water. Best overlaid structural poses for **A** with water positioned for production of **6a** (orange) or **6b** (purple) in AgAS:A723T with side chain hydroxyl constrained for deprotonation at C7 to produce **7**.

To investigate the hypothesis that substitution of serine or threonine for A723 in AgAS results in use of the introduced hydroxyl group to directly deprotonate **A**, the more selective A723T variant was investigated by the previously described *TerDockin* computational approach [3,28,29]. While the ability of unactivated hydroxyl groups to deprotonate such carbocations has been well established [30], modeling of AgAS:A723T indicates activation by the dipole moment of the G1 helix, as evidenced from the hydrogen bond to the carbonyl of the preceding residue (I722; see Figure 4). Much as was done with BjKS [3], each possible deprotonation site around the carbocation in **A** was investigated. Accordingly, distance and angle constraints between the threonine hydroxyl group and each of the five protons on the carbons flanking the carbocation (two each on C7 and C14 and one on C9), to enable deprotonation, were separately generated (e.g., Supplementary Figure S17a). In addition, the vinyl methylene was separately constrained to either end of the pyrophosphate co-product, reflecting the ionized ester bond and ensuring the reactant is correctly positioned within the active site. Thus, a total of ten constraint sets were generated and used to dock **A** within the modeled AgAS:A723T mutant (Supplementary Figure S17b) [3,29]. For each set, 4000 poses were generated (40,000 total). After filtering for those satisfying the constraints and exhibiting the most favorable total and interface energies, the remaining poses were sorted by which of the 3 possible olefinic isomers would be formed (i.e., **7**, **8**, or **10**). However, this indicated a strong preference for production of **8** (38/44), rather than the actually observed **7** (6/44; note no poses for **10** were left). Visual inspection of the binding orientations of the passing structures revealed substantial variation in positioning of the reactant **A** (Supplementary Figure S18). As control of the carbocation cascade is highly dependent on substrate positioning, it is unlikely that such large-scale change in the positioning would be observed [31], particularly given that threonine substitution for alanine decreases space in the active site. While the variation in orientation of **A** observed here might also reflect the use of the only available apo-enzyme structure [17], the AgAS active site must also contain a water that reacts with the rearranged carbocation intermediate abieta-8(14)-en-13-yl^+^ (**B**) to produce **6**, which might help constrain the positioning of the hydrocarbon reactant.

To model the reactant water, constraints were generated for such addition, first for the primary product, abieta-8(14)-en-13α-ol (**6a**), entailing distance and angles relative to the correct face of the 13-yl^+^ in **B**. Given that AgAS also makes the epimeric **6b**, a separate set of constraints for addition to the opposite face of the 13-yl^+^ in **B** were also generated. The distance constraint was then loosened to reflect the non-reactive nature of the water with **A**, and the previous docking simulations were repeated with these additional constraints (Supplementary Table S3). When the water is constrained relative to **A** as required for the production of **6a**, after filtering and grouping as before, there is a significant increase in the proportion of passing poses leading to **7** (Supplementary Table S4), which now represent a majority (22/42). By contrast, constraining the water relative to **A** for the production of **6b** leads to a lesser, although still improved, effect, with an increased number of the filtered (energetically favorable) poses leading to **7** (9/26). Notably, the positioning of **A** does not necessarily vary with the reactant water, consistent with the predominant production of the epimers of **6** by WT AgAS and, particularly, specific production of **7** by the A723T variant (Figure 4).

Notably, visual inspection of the passing poses revealed inconsistent placement of the water (and orientation of **A**) even for those leading to the observed product **7**. Thus, to find the appropriate position of the water, this was docked with **B** into the WT AgAS active site, generating 2500 poses, again with constraints from **B** to either end of the pyrophosphate co-product, for a total of 5000 poses for each epimer. When the water is constrained relative to **B** as required for production of **6a**, in the majority of the passing poses (5/9) the water sits near the pyrophosphate co-product (specifically nearest the bridging oxygen), which seems likely to serve as the catalytic base to deprotonate the alkyloxonium generated by addition of water. By contrast, in the other poses there is no evident functional group near the water that could serve this function. Similarly, when the water is constrained relative to **B** as required for production of **6b**, the only passing poses in which the water occupies a similar position (4/11) are also near the pyrophosphate co-product (albeit off one end), with the others also not generally adjacent to any potential catalytic base. Accordingly, in both cases the water is assumed to be proximal to the pyrophosphate co-product.

**Figure 5:**
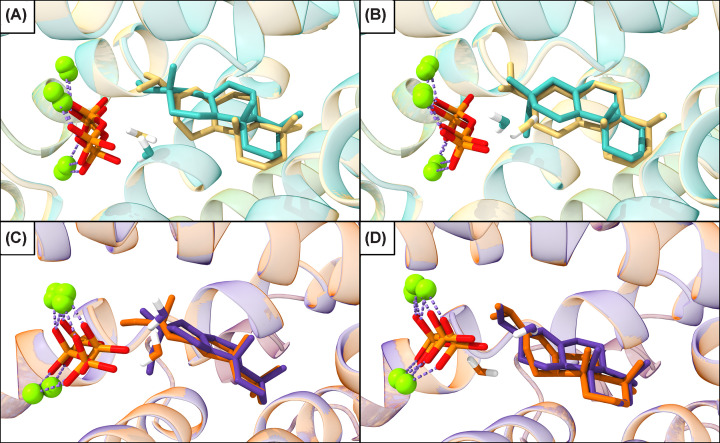
*TerDockin* results for AgAS. Overlaid best structural poses for (**A**) **A** (teal) and **B** (gold) with water positioned for production of **6a**; (**B**) **A** (teal) and **B** (gold) with water positioned for production of **6b**; (**C**) **A** with water positioned for either production of **6a** (orange) or **6b** (purple); and (**D**) **B** with water positioned for either production of **6a** (orange) or **6b** (purple).

Finally, to investigate the plausibility of the proposed mechanism, constraints were generated between C7 in the carbocation intermediates (**A** and **B**) and A723. These were added to docking simulations with WT AgAS along with the water—i.e., for addition to form either epimer of **6**. From the filtered poses, it was clear that the transition from **A** to **B** requires minimal movement of the reactant not only for each of the distinct water positions (Figure 5A,B), but even between these—e.g., between **A** in either case (Figure 5C), as well as **B** (Figure 5D). Thus, in the A723S/T mutants, the reactant water, presumably ligated by the pyrophosphate, is distant from C7 (where deprotonation of **A** would generate **7**) and then seems to serve in a structural role, as has been suggested for other terpene synthases [1,32].

## Conclusions

The results of the present study highlight the importance of the VSIAL motif in determining product outcome in Pinaceae diterpene synthases. Although previous work has identified the strong effect on product outcome for serine substitution of the alanine [14], which sits at a conserved helix break [17], none of the other variants explored here have been previously reported in the structurally defined AgAS. Of the hydroxyl introducing serine/threonine substitutions presented here, that of threonine for the isoleucine or alanine shows a stronger effect than substitution with serine. The influence of the single methyl group difference between these highlights the importance of precise substrate positioning in terpene synthase activity, much as previously shown in other terpene synthases [30]. While substitution of glycine for leucine might be viewed as contradictory, given its strong effect despite presumably increasing active site volume, this may be due to the presence of additional water(s) and/or other structural rearrangement, as has been postulated elsewhere for other terpene synthases [33]. This is presumably especially important when the enzymatic structure is not fully closed, as found here with AgAS.

Application of *TerDockin* to this system has enabled some insight into the parameters necessary for accurate application—i.e., the need to incorporate all reactants, including here water. The repeated application of this approach was enabled by its computationally inexpensive nature relative to the multiscale approaches taken elsewhere (e.g., [30]). Accordingly, it was then possible to define appropriate constraints to ensure consistent positioning of this water and then infer that the hydroxyl group introduced by the substitution of serine or, more specifically, threonine for A723 most likely acts directly as a catalytic base to deprotonate **A**. This highlights the importance of water in the diterpene synthase active site of AgAS, as previously hypothesized for other terpene synthases [1,32]. However, even this did not sufficiently limit the accessible space to fully reflect the nearly 100% product specificity observed with AgAS:A723T. Further work is required to improve the accuracy of *TerDockin* in this system. Nonetheless, the studies reported here both extend our understanding of active site interactions in the TPS-d3 subfamily and, hence, their evolution, as well as provide some insight into the accurate application of the *TerDockin* computational approach to understanding terpene synthase mechanisms.

## Methods

### General

All reagents were purchased from Fisher Scientific unless otherwise specified.

### Alignment of the TPS-d3 sub-family

Protein sequences of TPS-d3 sub-family members were those previously described [25]. These sequences were initially aligned using the MEGA12 software with the MUSCLE alignment tool [34]. For presentation, alignments were made using the Create Alignment tool (open gap cost—10 and gap extension cost = 1), which was used to generate a phylogenetic tree using the Create Tree (Neighbor Joining construction with Jukes-Cantor distance measurement and 1000 replicates) and rooted with the lycophyte LSs as the outgroup, using CLC Main Workbench (version 25.0.3; QIAGEN).

### Mutagenesis

A previously generated wild-type pseudomature AgAS pENTR Gateway system construct was used as the template for site-directed mutagenesis via PCR with overlapping mutagenic primers purchased from IDT [14]. Resulting mutant constructs were verified by sequencing. Wild-type and mutant genes were transferred by Gateway Cloning into the Gateway vector pDEST14.

### Metabolic engineering and product analysis

The genes were expressed in a previously described metabolic engineering system [26]. The recombinant *E. coli* strains were grown and extracted with hexanes before analysis by GC:MS as previously described [35]. Products were identified by comparison of retention times and mass spectra to authentic standards (Supplementary Figure S2).

### TerDockin analysis

Analysis of enzymatic activity was performed using the previously described *TerDockin* system [3,28,29]. *TerDockin* takes a chemically informed, molecular mechanic-based approach to accurately docking preoptimized conformer libraries into terpene synthases. To generate the conformer libraries for docking, the carbocation intermediates were first optimized using GAUSSIAN with the B3LYP functional with the 6-31+G(d,p) basis set. Using CREST, conformers were generated from this optimized structure, which were then also optimized as above. The conformers were filtered to remove duplicates as well as conformers with energies more than 5 kcal from the minimum. These were then further prepared as previously described [29]. Params files describing the water and pyrophosphate magnesium cluster were used from previous work. For docking, a previously described crystal structure of AgAS (PDB: 3S9V) was relaxed using the Rosetta FastRelax protocol. Initial docking constraints and chemical constraints used are those previously described [3]. Additional constraints for the introduction and positioning of the water are described in Supplementary Table S3. To correctly position the pyrophosphate-magnesium cluster and water, an additional grid-based transformer was included. For each constraint set 4000 structures were generated and pooled, yielding 40,000 structures in the case of deprotonation site prediction or 8000 in the case of wild-type docking. These poses were filtered such that the individual constraint scores for each ligand were below one, ensuring constraint fulfillment. These were then filtered to leave only structures with total scores within the lowest 10%, followed by those with carbocation interface energies within the lowest 10%.

## Supplementary Material

Supplementary Figures S1-S18 and Tables S1-S4

## Data Availability

The structural data from the *TerDockin* analyses has been deposited and is publicly accessible at https://doi.10.25380/iastate.30688517 [36]. Accession code for AgAS: AAB05407.

## Open Access

Open access for this article was enabled by the participation of Iowa State University in an all-inclusive Read & Publish agreement with Portland Press and the Biochemical Society.

## Abbreviations

AgAS: *Abies grandis*
AS: abietaenol synthase
CPP: copalyl pyrophosphate
GGPP: geranylgeranyl pyrophosphate
LS: levopimaradiene synthase
PaPS: *Picea abies*
PS: (iso)pimaradiene synthase
