# Correction: Docking in the Abies grandis abietaenol synthase to investigate the mechanism underlying conifer resin acid diterpene synthase evolution

**DOI:** 10.1042/BCJ20250294_COR

**Published:** 2026-08-12

**Authors:** Mark Schmidt-Dannert, Griffin Humphreys, Taylor Eich, Meirong Jia, Reuben J. Peters

**Affiliations:** 1 Roy J. Carver Department of Biochemistry, Biophysics & Molecular Biology, Iowa State University, Ames, IA 50011, U.S.A.; 2 State Key Laboratory of Bioactive Substance and Function of Natural Medicines, NHC Key Laboratory of Natural Products, CAMS Key Laboratory of Enzyme and Biocatalysis of Natural Drugs, Institute of Materia Medica, Chinese Academy of Medical Sciences and Peking Union Medical College, Beijing 100050, China

**Keywords:** diterpenoid, enzymology, evolutionary biology

The authors of the original article “Docking in the Abies grandis abietaenol synthase to investigate the mechanism underlying conifer resin acid diterpene synthase evolution” (DOI: **10.1042/BCJ20250294**) would like to correct Figure 3.

During the proofing process, an incorrect version of Figure 3 was mistakenly included by the authors. The authors would therefore like to replace this image with the correct version of Figure 3.

The requested correction has been assessed and agreed to by the Editorial Board. The authors declare that these corrections do not change the results or conclusions of their paper.

The corrected version of Figure 3 is presented here.

**Figure 3:**
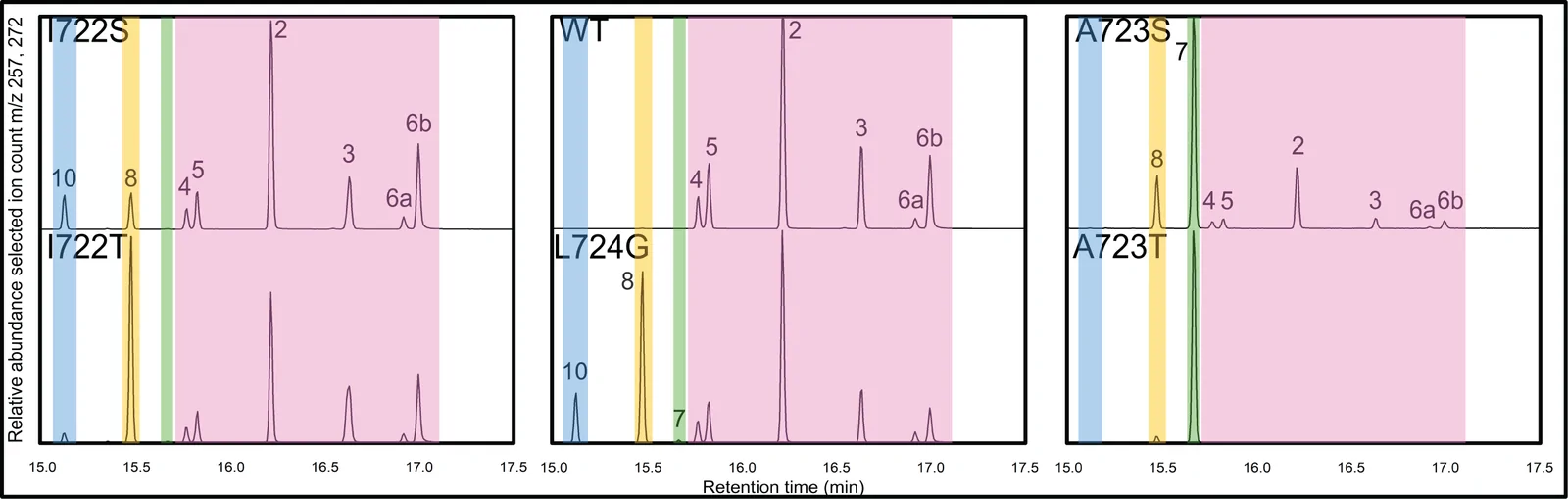
Effect of phylogenetically guided subsitution for VSIAL motif in AgAS

