# Supplementary material for: Docking in the Abies grandis abietaenol synthase to investigate the mechanism underlying conifer resin acid diterpene synthase evolution
Source: Biochem J. 2026 Feb 24;483(3):365–74. doi: 10.1042/BCJ20250294 (PMC13089139; doi:10.1042/BCJ20250294)
Supplement: Supplementary Figures S1-S18 and Tables S1-S4 [file BCJ-2025-0294_supp.pdf]

Supplemental Information for:

Docking in the *Abies grandis* abietaenol synthase to investigate the mechanism underlying conifer resin acid diterpene synthase evolution

Mark Schmidt-Dannert,[1] Griffin Humphreys,[1] Taylor Eich,[1] Meirong Jia[2,*] and Reuben J. Peters[1,*]

[1]Roy J. Carver Department of Biochemistry, Biophysics & Molecular Biology, Iowa State University, Ames, IA 50011, USA

[2]State Key Laboratory of Bioactive Substance and Function of Natural Medicines, NHC Key Laboratory of Natural Products, CAMS Key Laboratory of Enzyme and Biocatalysis of Natural Drugs, Institute of Materia Medica, Chinese Academy of Medical Sciences and Peking Union Medical College, Beijing 100050, China

*Corresponding authors: Meirong Jia (email: jiameirong@imm.ac.cn)

Reuben J. Peters (email: rjpeters@iastate.edu)

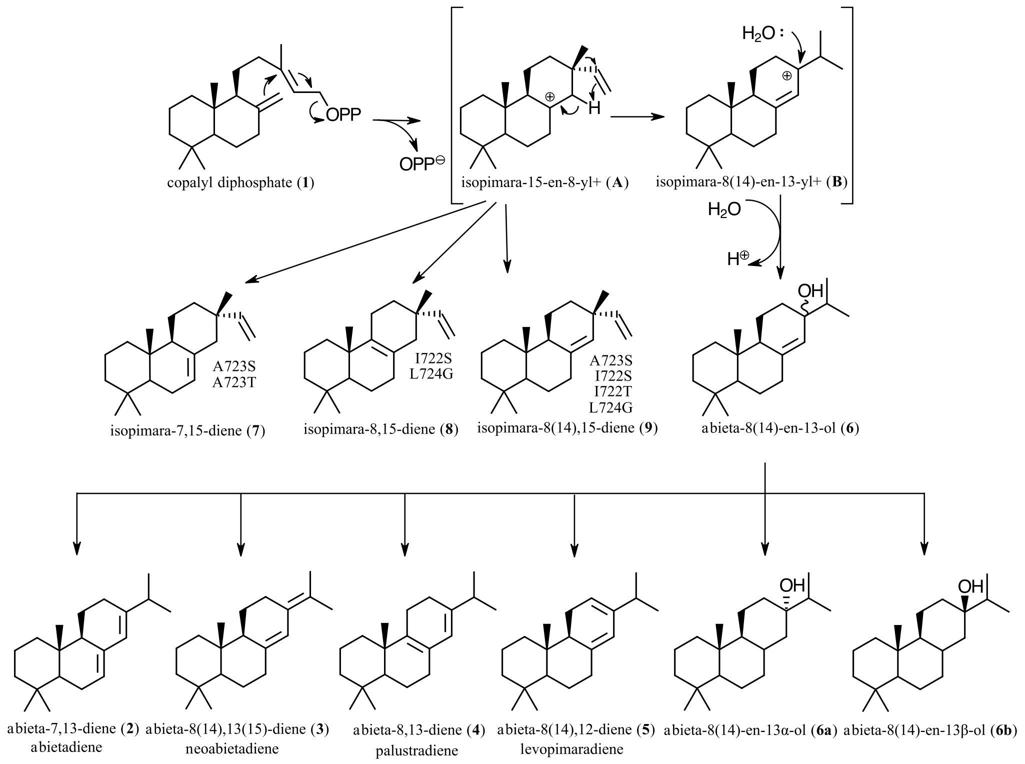

Figure S1: AgAS DTS reaction and observed products (including olefins derived from dehydration of **6** with common names indicated below).

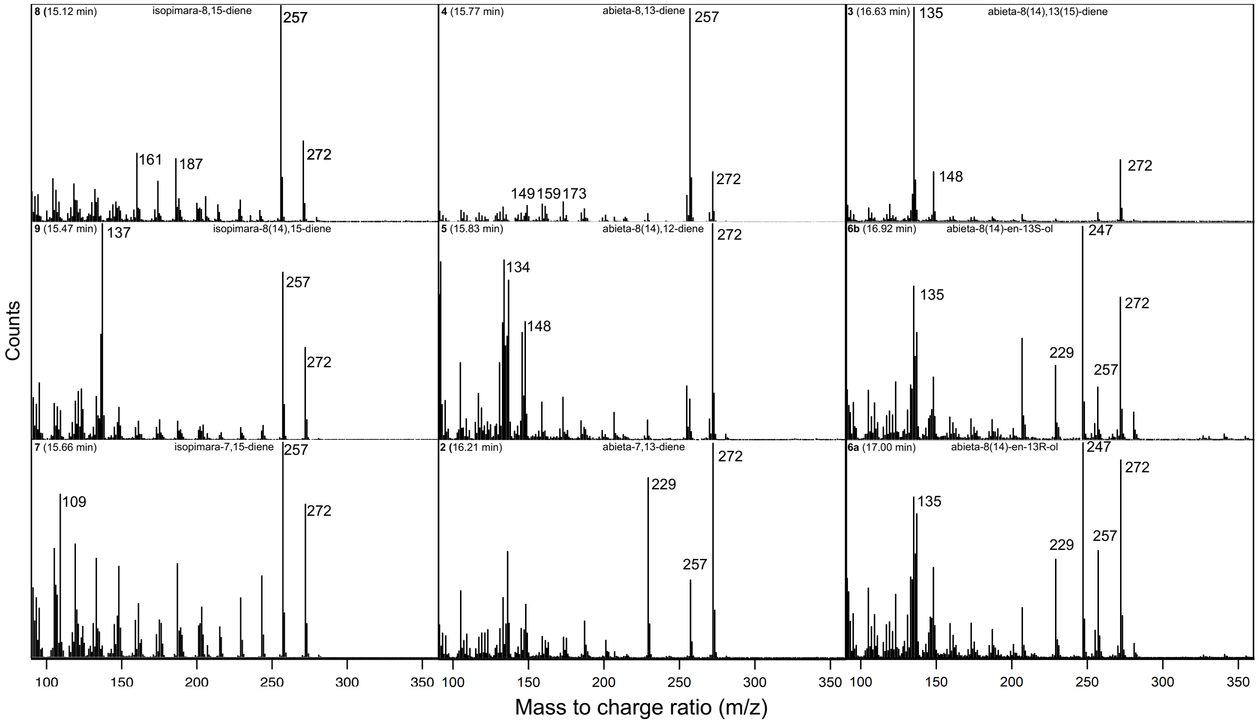

Figure S2: Mass spectra of selected products from conifer diterpene synthases.

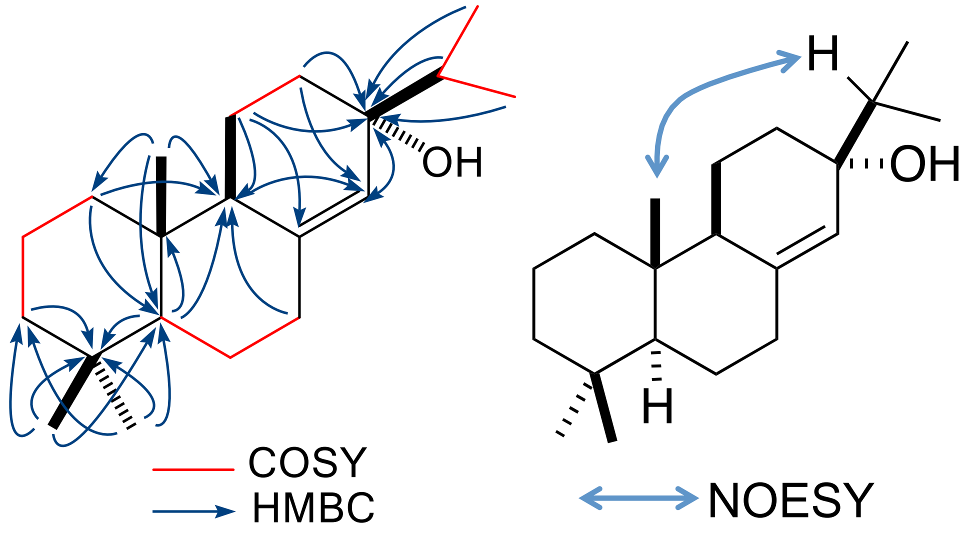

Figure S3: COSY, HMBC, and NOESY correlations observed for abieta-8(14)-en-13α-ol

Table S1: ^{1}H-NMR and ^{13}C-NMR assignments for abieta-8(14)-en-13α-ol (solvent: benzene-d_6)

POSITION	Δ_C (PPM)	Δ_H (PPM)	MULTIPLICITY
**1**	39.3	0.85	m
		1.54	m
**2**	19.4	1.48	m
		0.87	m
**3**	42.4	1.13	m
		1.38	m
**4**	33.4	-	
**5**	55.2	0.93	m
**6**	23.2	1.99	m
		1.21	m
**7**	36.4	β 2.21	m
		α 1.98	td (J = 13.6, 5.5 Hz)
**8**	139.1	-	
**9**	50.7	1.57	m
**10**	39.0	-	
**11**	19.1	1.51	m
		1.26	m
**12**	30.2	1.86	m
		1.23	m
**13**	71.9	-	
**14**	129.3	5.47	s
**15**	36.4	1.78	m
**16**	17.1	1.09	d (J = 6.7 Hz)
**17**	17.3	1.00	d (J = 6.9 Hz)
**18**	33.9	0.86	s
**19**	22.2	0.82	s
**20**	15.1	0.72	s

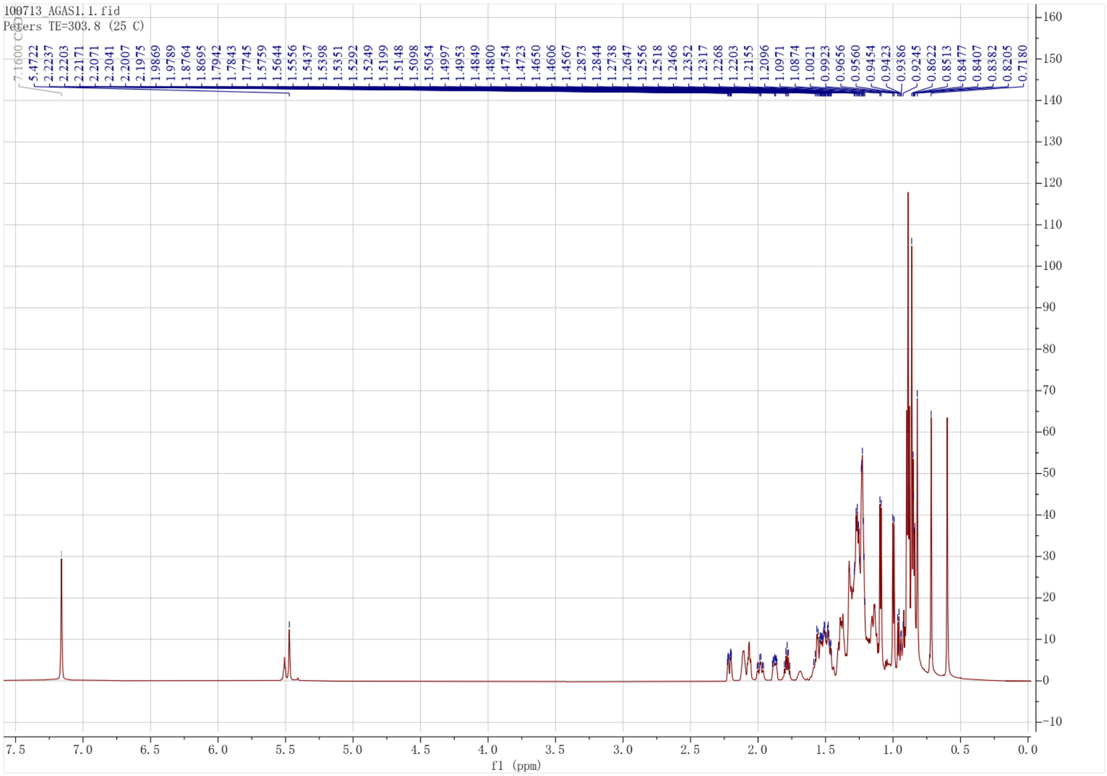

Figure S4: [1]H Spectrum of abieta-8(14)-en-13α-ol

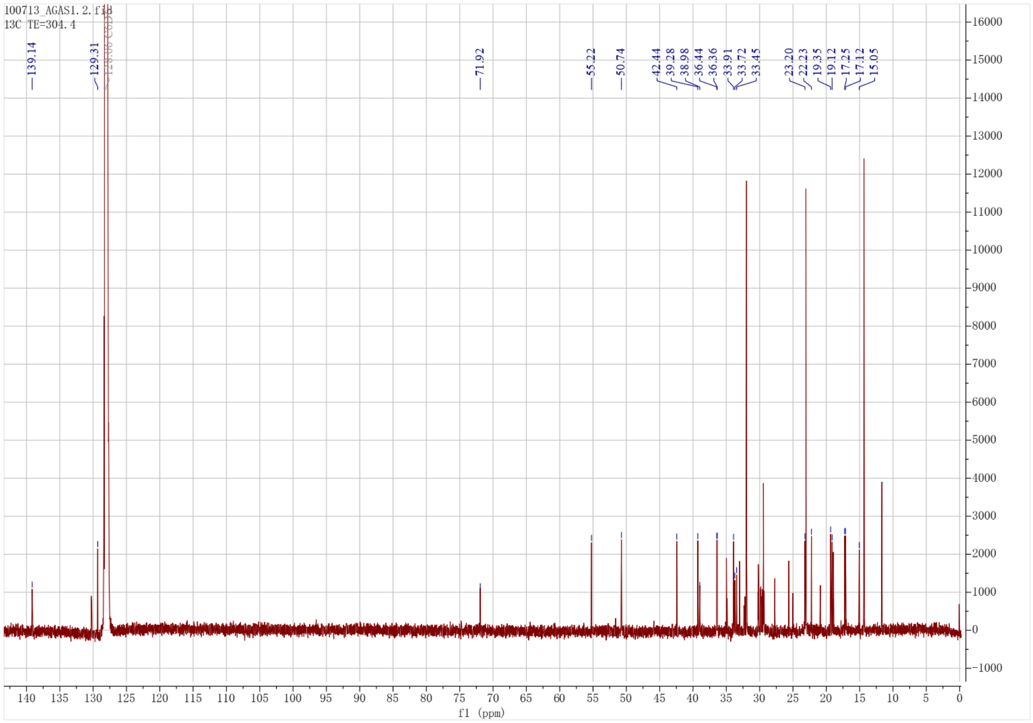

Figure S5: ^{13}C spectrum of abieta-8(14)-en-13α-ol

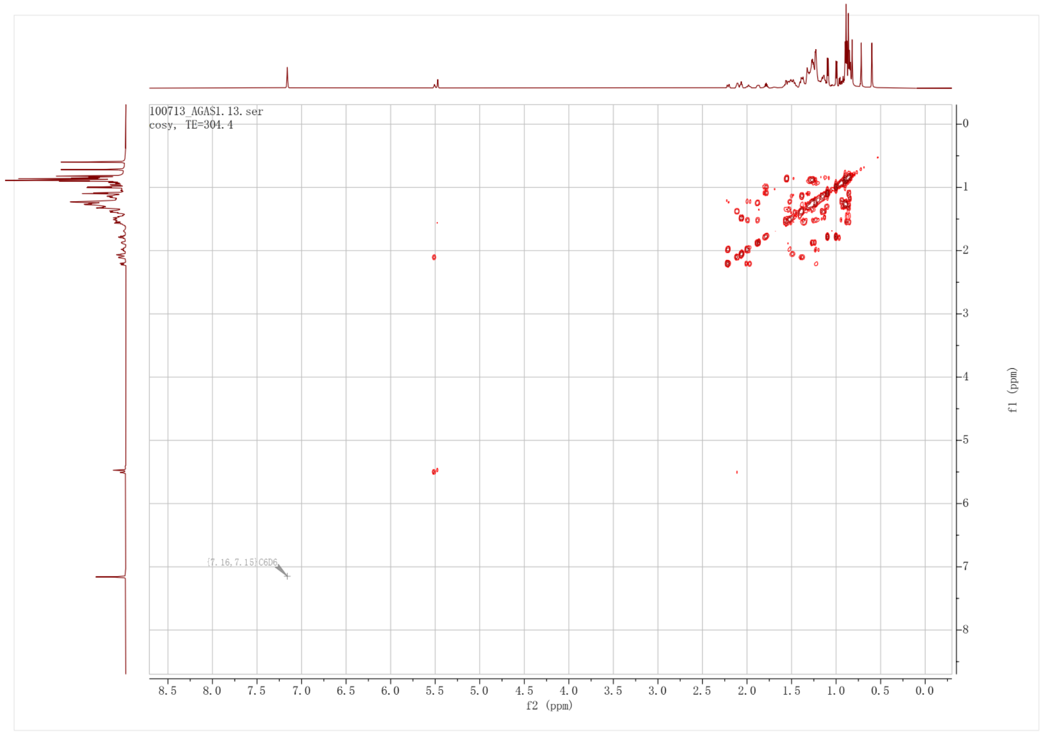

Figure S6: ^{1}H-^{1}H COSY Spectrum of abieta-8(14)-en-13α-ol

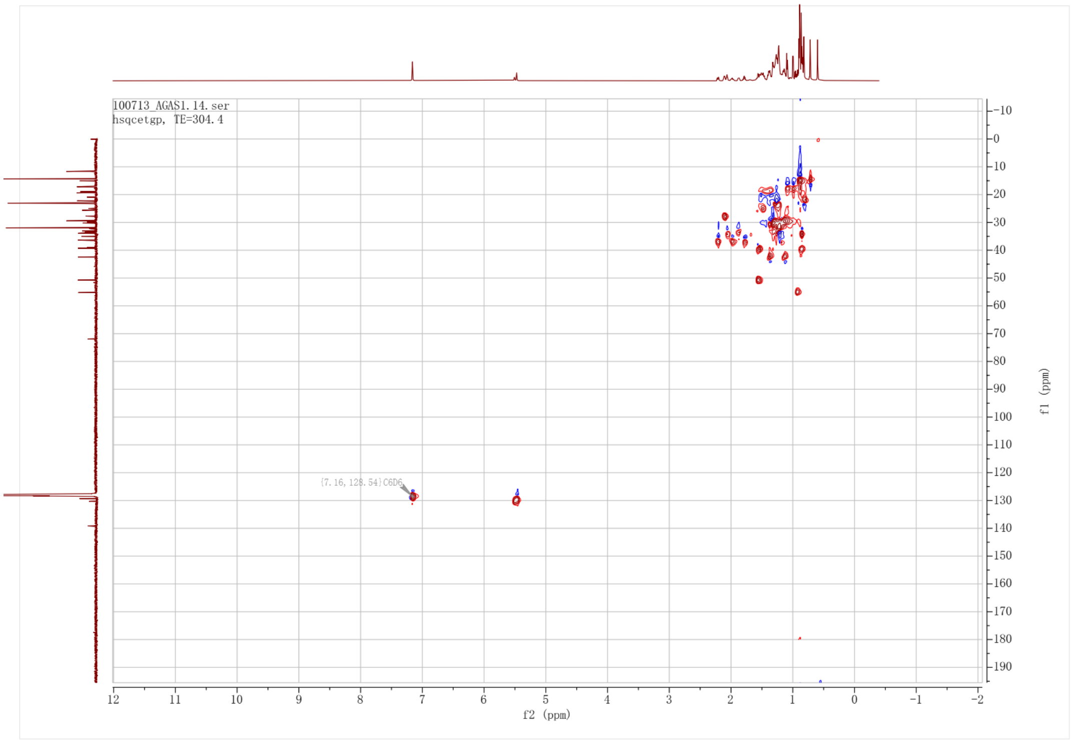

Figure S7: HSQC Spectrum of abieta-8(14)-en-13α-ol

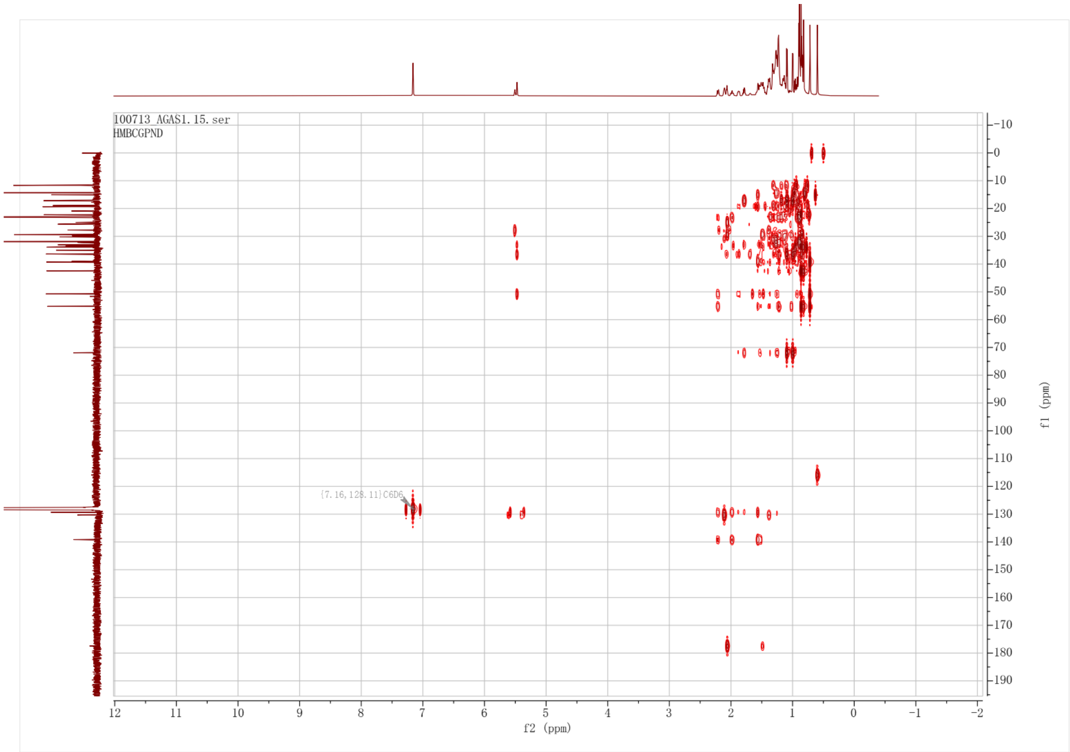

Figure S8: HMBC spectrum of abieta-8(14)-en-13α-ol

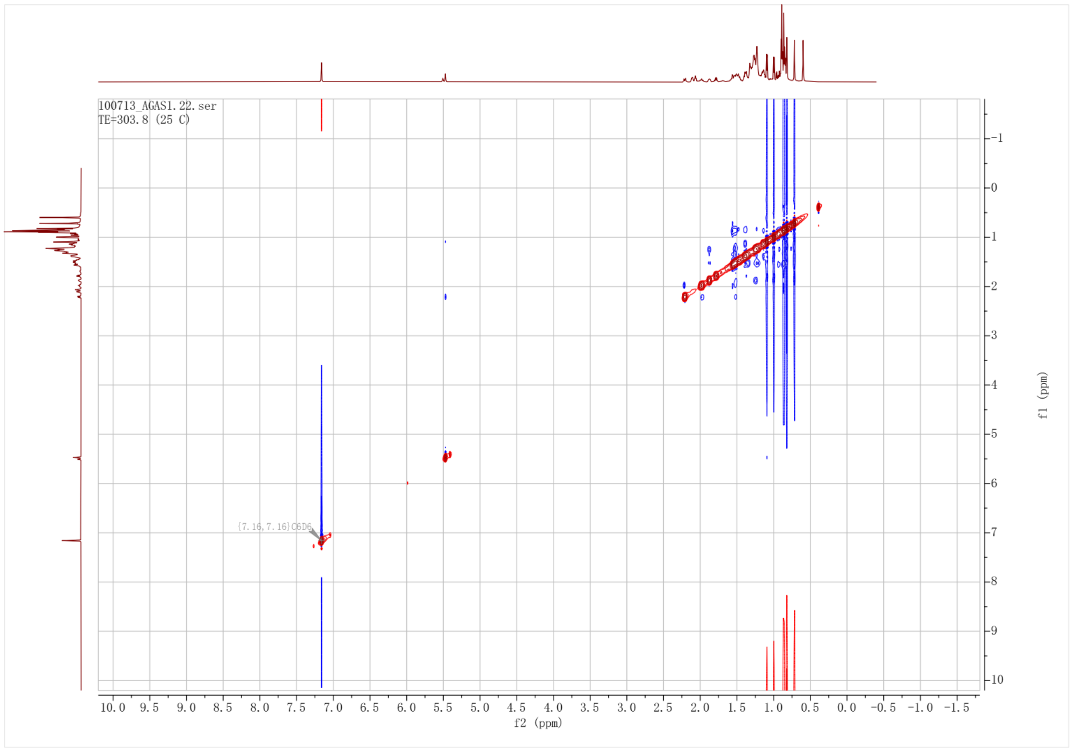

Figure S9: NOESY spectrum of abieta-8(14)-en-13α-ol

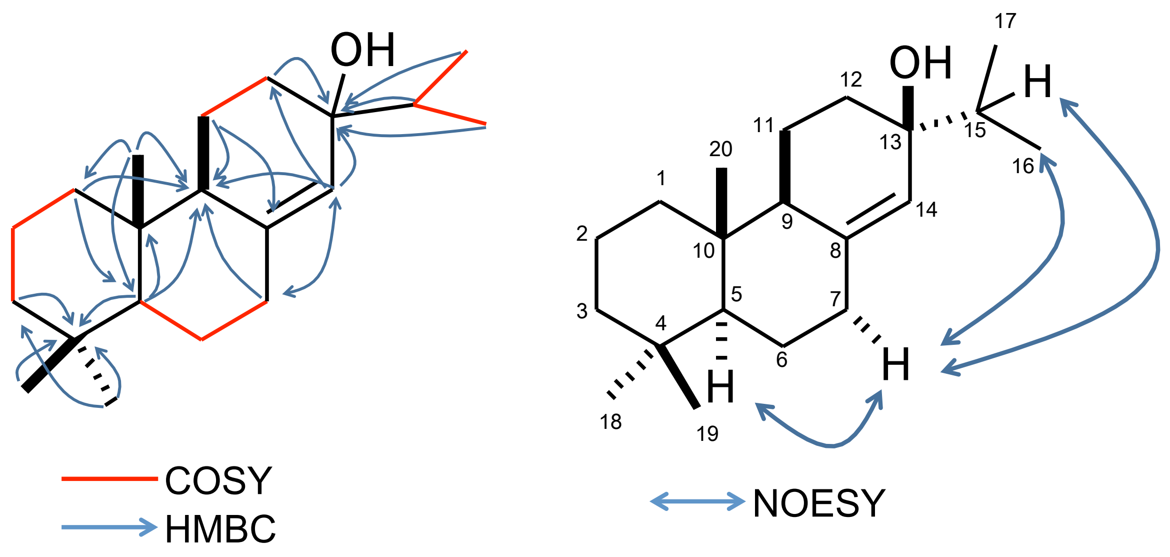

Figure S10: COSY, HMBC, and NOESY correlations observed for abieta-8(14)-en-13β-ol

Table S2: NMR Assignments of abieta-8(14)-en-13β-ol (solvent: benzene-d_6)

POSITION	Δ_C (PPM)	Δ_H (PPM)	MULTIPLICITY
**1**	39.5	0.89	m
		1.60	m
**2**	19.3	1.46	m
		0.84	m
**3**	42.4	1.13	m
		1.38	m
**4**	33.3	-	
**5**	54.6	0.90	m
**6**	22.6	1.47	m
		1.23	m
**7**	35.7	β 2.19	brd (J = 13.8 Hz)
		α 1.95	dd (J = 13.8, 4.5 Hz)
**8**	142.4	-	
**9**	51.6	1.51	m
**10**	38.1	-	
**11**	18.0	1.61	m
		1.50	m
**12**	30.4	1.64	m
		1.29	m
**13**	71.2	-	
**14**	127.3	5.40	s
**15**	38.6	1.72	qq (J = 6.5, 6.5 Hz)
**16**	16.9	1.04	d (J = 6.5 Hz)
**17**	17.7	0.94	d (J = 6.5 Hz)
**18**	33.9	0.85	s
**19**	22.2	0.82	s
**20**	14.7	0.72	s

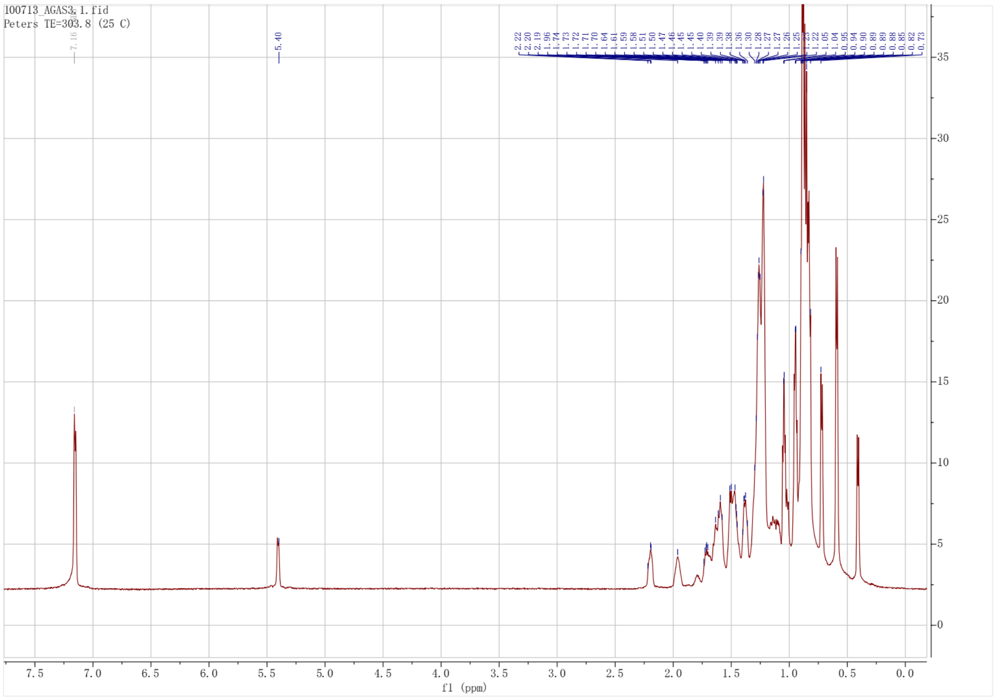

Figure S11: ^{1}H spectrum of abieta-8(14)-en-13β-ol

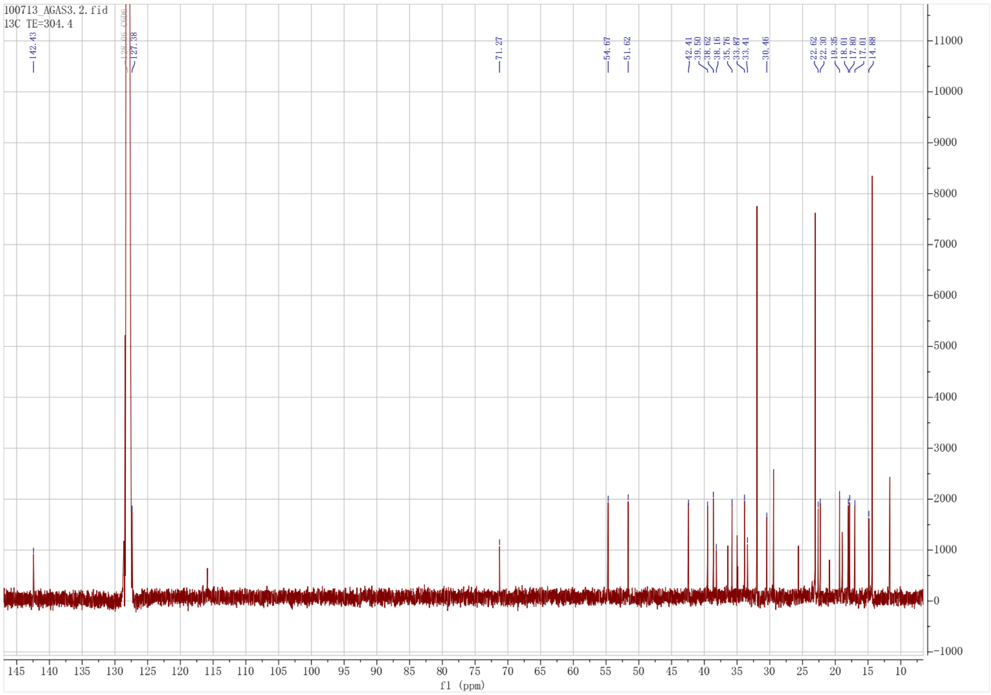

Figure S12: ^{13}C spectrum of abieta-8(14)-en-13β-ol

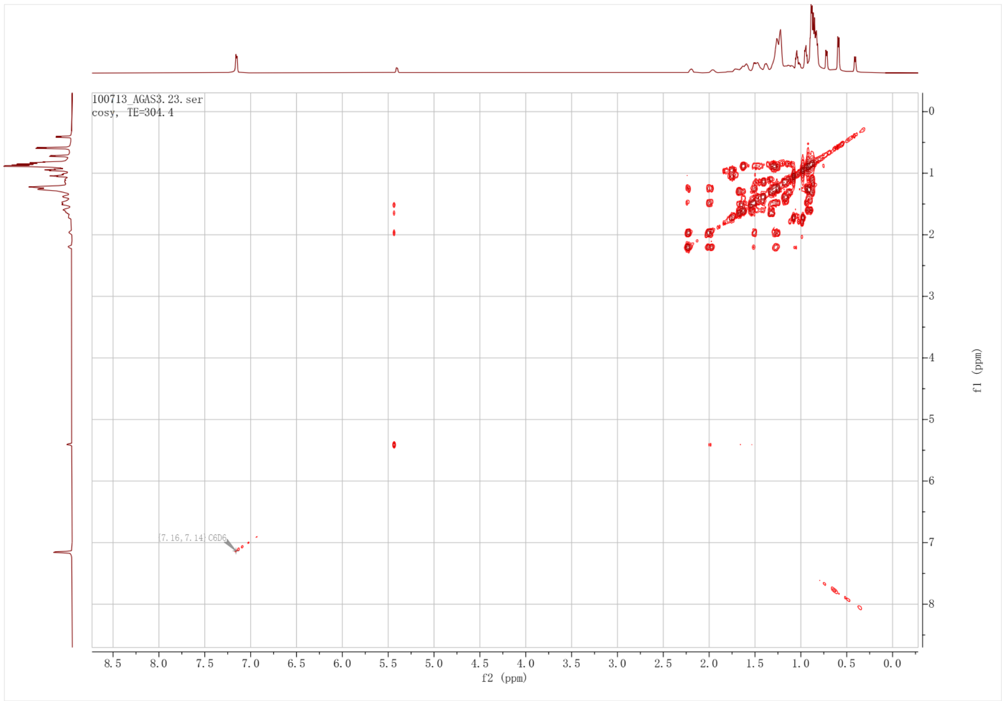

Figure S13: ^{1}H-^{1}H COSY spectrum of abieta-8(14)-en-13β-ol

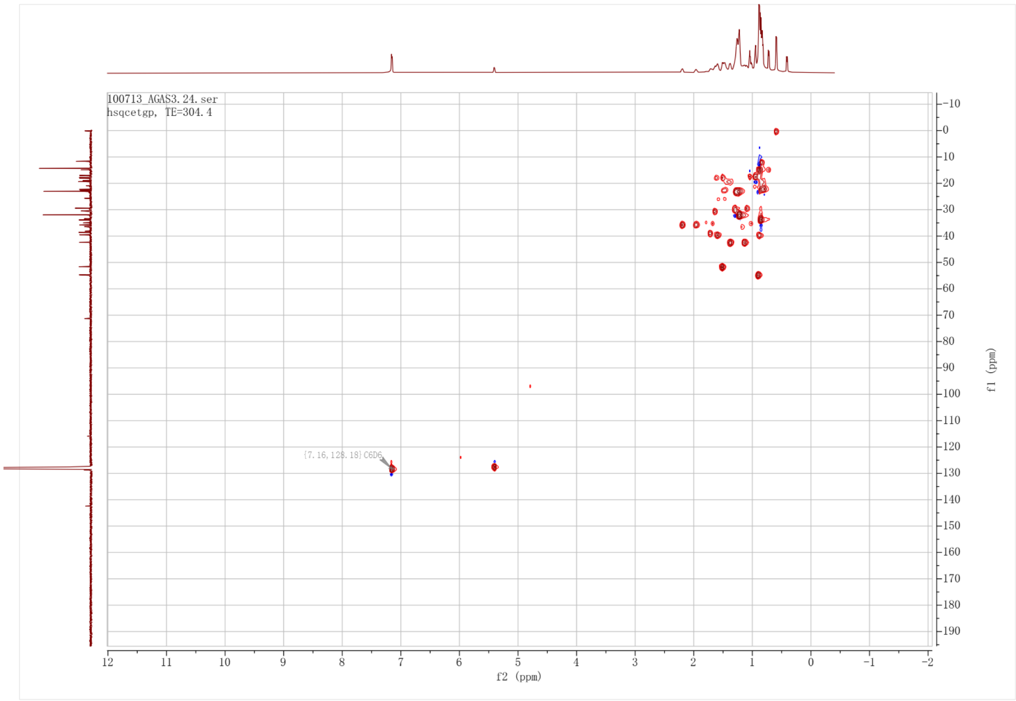

Figure S14: HSQC spectrum of abieta-8(14)-en-13β-ol

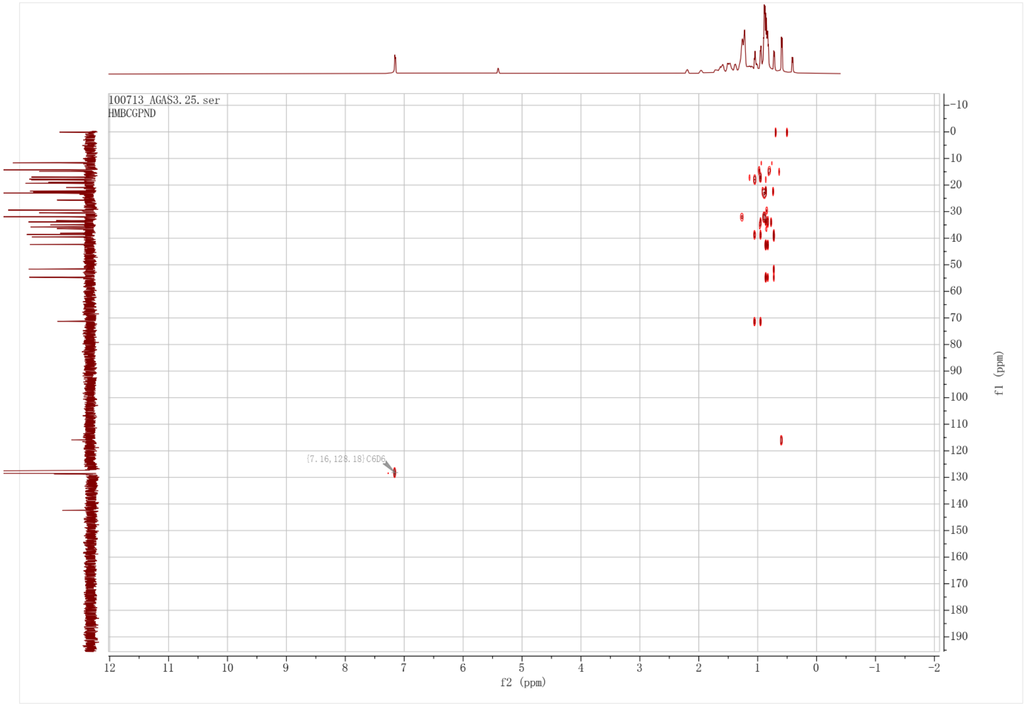

Figure S15: HMBC spectrum of abieta-8(14)-en-13β-ol

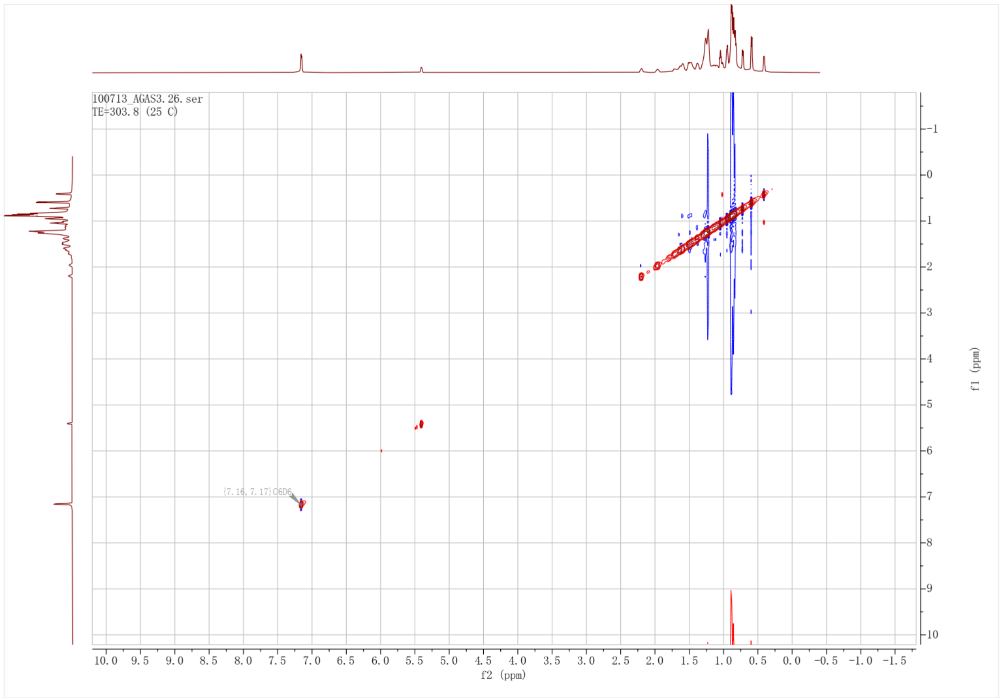

Figure S16: ^{1}H-^{1}H NOESY spectrum of abieta-8(14)-en-13β-ol

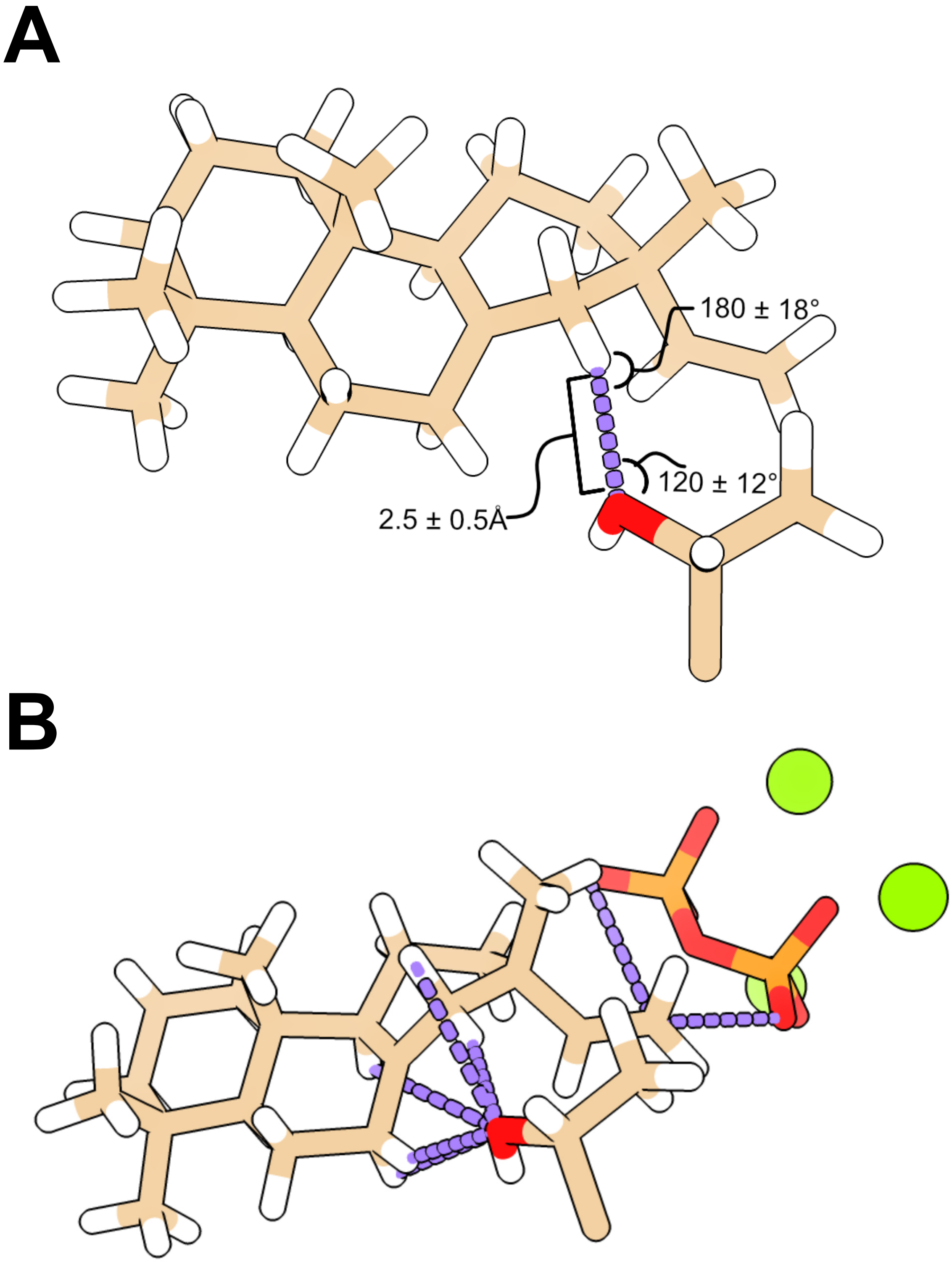

Figure S17: **A)** Distance and angle constraints for deprotonation of **A. B)** All constraints used in docking of AgAS

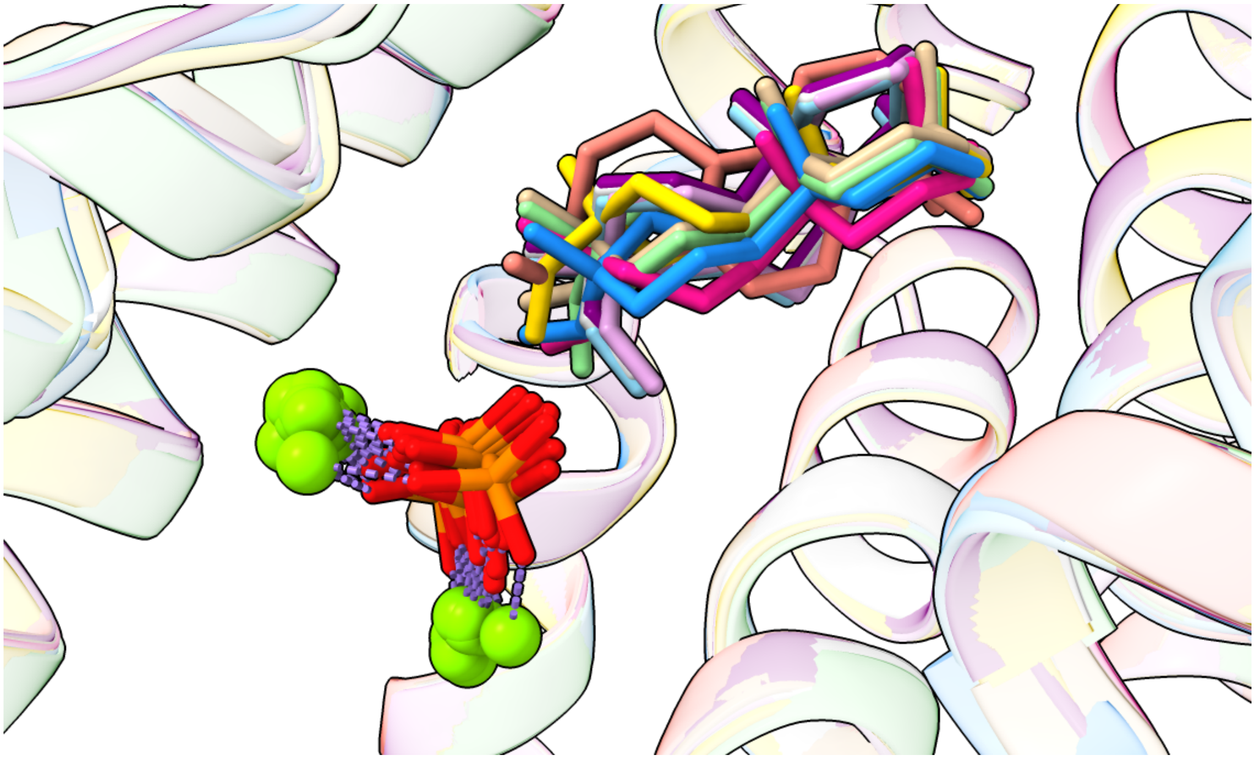

Figure S18. Binding orientations observed for passing poses of structures with **A** docked constrained only for deprotonation.

Table S3: Constraints included for the positioning of water relative to carbon 13

	**alpha addition**	
	**intA**	**intB**
**Distance**	3.00 ± 1.5	3.00 ± 0.5
**Angle**	110 ± 15	110 ± 15
**Torsion**	90 ± 75	90 ± 75
	**beta addition**	
	**intA**	**intB**
**Distance**	3.00 ± 1.5	3.00 ± 0.5
**Angle**	110 ± 15	110 ± 15
**Torsion**	270 ± 75	270 ± 75

Table S4: Number of passing poses for each constraint set grouped by product, abstracted proton, and pyrophosphate orientation. **A)** Constraint set modelling only deprotonation of the proton. **B)** Constraint sets including both water to carbocation and deprotonation constraints. **C)** Constraint sets including water to pyrophosphate, water to carbocation, and deprotonation constraints.

**A)**	C7 Orientation 1		C14 Orientation 1		C9 Orientation 1
Deprotonated H	H17	H18	H26	H27	H14
Poses	0	1	6	19	0
	C7 Orientation 2		C14 Orientation 2		C9 Orientation 2
Deprotonated H	H17	H18	H26	H27	H14
Poses	4	1	9	4	0
SUM	6		38		0
**B)**	C7 Orientation 1		C14 Orientation 1		C9 Orientation 1
Deprotonated H	H17	H18	H26	H27	H14
Poses	9	4	4	8	0
	C7 Orientation 2		C14 Orientation 2		C9 Orientation 2
Deprotonated H	H17	H18	H26	H27	H14
Poses	7	2	4	4	0
SUM	22		20		0
**C)**	C7 Orientation 1		C14 Orientation 1		C9 Orientation 1
Deprotonated H	H17	H18	H26	H27	H14
Poses	6	0	8	4	0
	C7 Orientation 2		C14 Orientation 2		C9 Orientation 2
Deprotonated H	H17	H18	H26	H27	H14
Poses	3	0	5	0	0
SUM	9		17		0
